# Improvement in the Synthesis Conditions and Studying the Physicochemical Properties of the Zeolite Li-A(BW) Obtained from a Kaolinitic Rock

**DOI:** 10.1038/s41598-020-62702-8

**Published:** 2020-03-31

**Authors:** Daniela Novembre, Domingo Gimeno, Alessandro Del Vecchio

**Affiliations:** 1 Dipartimento di Ingegneria e Geologia, Università di Chieti-Pescara, Via dei Vestini 30, 66013 Chieti, Italy; 2 Department Mineralogia, Petrologia i Geologia Aplicada, Universitat de Barcelona, 08028 Barcelona, Spain

**Keywords:** Mineralogy, Characterization and analytical techniques

## Abstract

Crystallization of zeolite Li-A(BW) from kaolinite (Standard Porcelain by the IMERYS Minerals Ltd) through a conventional hydrothermal treatment is here achieved for the first time with no additives as reported in the literature. Moreover lower kaolin calcination temperatures and lower synthesis temperatures are tested and verified in this work. The synthesis process is rather simple as the reaction of kaolinite with alkali occurs very readily after calcination of at 650 °C. Metakaolin is mixed with calculated amount of aluminum hydroxide and lithium hydroxide and the experiment is performed at ambient pressure and 180 ± 0.1 °C. Li-A(BW) is characterized by powder X-ray diffraction, high temperature X-ray diffraction, scanning electron microscopy, inductively coupled plasma optical emission spectrometry, thermal analysis and infrared spectroscopy. Calculation of cell parameters (through Rietveld Refinement) and density, specific surface and pore size are also achieved. The amount of amorphous phase in the synthesis powders is estimated with quantitative phase analysis using the combined Rietveld and reference intensity ratio methods. The results become notably attractive in view of a possible industrial transfer of the synthesis protocol.

## Introduction

Zeolites are a group of tectosilicates of about 50 minerals with synthetic analogues. Their structure is made of three-dimensional networks of Al/Si tetrahedra arranged to form channels containing water and exchangeable alkaline or alkaline earth cations.

Zeolite Li-A(BW) is a synthetic low silica zeolite, which possesses the ABW framework topology. Low silica zeolites such as Li-A(BW), are widely involved in several technological applications, such as ion exchangers, adsorbents and catalysts, in radioactive-waste water treatments, sewage effluent treatments, agricultural-waste water treatments, as materials for ferroelectric devices^1^.

Zeolite Li-A(BW) was first synthesized by Barrer and White^2^ and the framework structure and water positions were later determined by Kerr^3^ by X-ray powder diffraction (*Pna2*_1_, *a* = 10.31, *b* = 8.18; *c* = 5.00 Å). Krogh Andersen & Ploug-Sorensen^4^ confirmed this structure by X-ray single crystal refinement and later Norby *et al*.^5^ gave the lithium and hydrogen positions by neutron powder diffraction. From the above structural investigations, it results a framework characterized by 4-, 6-, and 8-rings of TO_4_ tetrahedra and a fully ordered Si/Al distribution. The result is a zig-zag chain of 4-rings running along the *c-* axis. These chains are linked together, forming 8-ring channels, in which water molecules and lithium ions are situated.

Li-A(BW) is characterized by limited reversible rehydration^5^ and undergoes to a displacive transition collapsing into the anhydrous phase γ-eucriptite at 650 °C^6^. The response to compression of Li-A(BW) zeolite was explored by synchrotron X-ray powder diffraction experiments in the range *P*_amb_-8.9 GPa resulting in a cell volume decrease of 12%^7^.

Synthesis of Li-A(BW) zeolite was performed in the past by the use of lithium hydroxide, aluminum hydroxide ad silica gel^2,8^; Andrade *et al*.^9^ added also a tetramethylammonium hydroxide as structure directing agent. Norby *et al*.^6^ and Dong *et al*.^10^ prepared the precursors gels using zeolite NaA and lithium chloride.

The large demand for low silica zeolites, due to their great technological potential, has led researchers to look for less expensive starting materials in order to obtain economically beneficial industrial results. In the light of reducing synthesis costs Yao *et al*.^11^ synthesized Li-A(BW) by fusion method using fly ash as raw material.

Another attempt at synthesis of zeolite Li-A(BW) was carried out starting from kaolinite^12^. Among clay minerals, in fact, Kaolinite is the most common phyllosilicate involved in successful zeolitic synthesis because of its particularly ample supply and availability and the well-known reactivity of thermally treated kaolin clays (metakaolin) with alkali^13–17^.

Lin *et al*.^12^ synthesized JBW, CAN, SOD and Li-A(BW) by the hydrothermal transformation of China meta-kaolin in the presence of inorganic additives. Synthesis of Li-A(BW) was operated starting from meta-kaolin and lithium hydroxide; optimal condition for the synthesis were: calcination temperature of kaolin, 800 °C, synthesis temperature of 200 °C, reaction period of 96 h and molar composition in reactant of 1Al_2_O_3_-2SiO_2_-1.5Li_2_O-31H_2_O.

In this paper we present the results of a research carried out to define the most favorable conditions for the synthesis of zeolite Li-A(BW) from metakaolin. The aim is to improve previous mineral synthesis attempts starting from kaolinite; i.e. by developing a synthesis protocol that does not include the use of additives and secondly working on the reduction of calcination and synthesis temperatures and on the reduction of synthesis times. Here for the first time an in-depth physico-chemical characterization of zeolite Li-A(BW) synthesized from meta-kaolinite is reported. Moreover, the synthesis of minerals starting from natural rocks can lead to final products that can be characterized by various degrees of impurities^12^ deriving from the starting material in fact used. For this purpose, in this work the degree of purity of the synthesized zeolite is defined through a quantitative phase analysis using the combined Rietveld and reference intensity ratio methods.

## Materials and Methods

The kaolin sample used in the present study is Standard Porcelain from IMERYS MINERALS LTD (Cornwall, UK). For the chemical composition of kaolin and its mineralogical, morphological and spectroscopic characterization, see Novembre *et al*.^16,18^. Preliminary calcination of kaolin was carried out using the following procedure: aliquots of kaolin were placed in open porcelain crucibles which were heated in a Gefran Model 1200 furnace (GEFRAN SPA, Brescia, Italy) to the calcination temperature of 650 °C ±1° at a pressure of 1 atm. The heating rate of the sample was 1.5 °C s^−1^. Once the calcination temperature was reached, the crucibles were left in the furnace for 2 h and then removed and cooled at room temperature.

The Al(OH)_3_ and LiOH pellets used in the synthesis protocol were purchased from HONEYWELL RIEDEL-DE HAËN (HONEYWELL RIEDEL-DE HAËN, Bucharest, Romania). The purity of the reagents was of 99%.

1.35 g of LiOH pellets have been dissolved in 22 ml of distilled water. 0.8 g of Al(OH)3 (65%, p/v) and 3.10 g of kaolin were mixed with the LiOH solution. The initial mixture had the composition: 1.75 SiO_2_-1 Al_2_O_3_-1,75 LiO_2_-1,26 H_2_O.

The mixture was homogenized for two hours with a magnetic stirrer. Then was put inside a stainless-steel hydrothermal reactor and heated at 10 °C/min until 190 °C and kept for 2, 6, 15, 30 and 140 h. Synthesis products were sampled periodically from the reactor, filtered with distilled water and dried in an oven at 40 °C for a day.

Kaolin and products of synthesis were analysed by powder X-ray diffraction (XRPD); the instrument was a SIEMENS D5000 operating with a Bragg-Brentano geometry (CuKα = 1.518 Å, 40 kV, 40 mA, 2–45°, 2–90° 2theta scanning interval, step size 0.020° 2theta). Identification of Li-A(BW) and relative peak assignment was performed with reference to the following JCPDS code: 00-041-0554.

Both the crystalline and amorphous phases in the synthesis powders were estimated using quantitative phase analysis (QPA) applying the combined Rietveld and reference intensity ratio (RIR) methods; corundum NIST 676a was added to each sample, amounting to 10%, and the powder mixtures were homogenized by hand-grinding in an agate mortar^19^. Data for the QPA refinement were collected in the angular range 5–120° 2theta with steps of 0.02° and 10 s step^−1^, a divergence slit of 0.5° and a receiving slit of 0.1 mm.

Data were processed with the GSAS software and the graphical interface EXPGUI^20,21^ starting with the structural models proposed by Krogh Andersen and Ploug-Sorensen^4^ for Li-A(BW). The following parameters were refined: background parameters, zero shift, cell parameters and peak profiles.

Morphological analyses were obtained by means of scanning electron microscopy (JEOL JSM-840 served by a LINK MICROANALYSIS EDS system, with operating conditions of 15 kV and window conditions ranging from18 to 22 mm)^22^.

Induced coupled plasma optical emission spectroscopy technique (ICP-OES, PERKIN ELMER OPTIMA 3200 RL) was performed on synthesized powders through previous fusion (Pt meltpot) in lithium meta-tetra borate pearls and subsequent acid solubilisation and analytical determination^23^.

Li-A(BW) density was calculated by He-picnometry using an ACCUPYC 1330 pycnometer. The specific surface and porosity were obtained by applying the BET (BRUNAUER-EMMETT-TELLER) method with N_2_ using a MICROMERITICS ASAP2010 instrument (operating from 10 to 127 kPa)^19^.

The infrared analysis was performed with a spectrometer FTLA2000, served by a separator of KBr and a DTGS detector; the source of IR radiation was a SiC (GLOBAR) filament. Samples were treated according the method of Novembre *et al*.^24^ using powder pressed pellets (KBr/sample ratio of 1/100, pressure undergone prior determination 15 t/cm^2^); spectra were processed with the program GRAMS-Al (THERMO SCIENTIFIC COMPANY).

Thermal stability and phase transformations were studied using high-temperature X-ray diffractometry with a PANALYTICAL X’PERT PRO MPD (CuKα = 1.518 A °, 45 kV, 40 mA, X’CELERATOR DETECTOR with active length of 2.122 °, θ/2θ scan from 5 to 50° 2θ with step size of 0.017° and measuring time of 100 seconds per step), equipped with a high temperature camera ANTON PAAR HTK1200N (thermocouple Pt 10% RhPt). The sample holder was a platform with a 16 mm diameter equipped with a ceramic cup (0.8 mm deep and 14 mm inner diameter) for holding powder. The analyses were taken at different temperatures: from 28° up to 1200 °C, every 100 °C. Slope was 10 °C/min. The program pakage GSAS - EXPGUI was used for the calculation of cell parameters, using the Rietveld full-profile method starting with the structural models proposed by Krogh Andersen and Ploug-Sorensen^4^ for Li-A(BW), and Norby^6^ for γ-Eucryptite and β-Eucryptite.

Differential thermal analysis (DTA) and thermogravimetry (TG) were performed on the zeolitic powder using a Mettler TGA/SDTA851e instrument (10°/min, 30_1100 °C, sample mass of ~10 mg, Al_2_O_3_ crucible) (METTLER TOLEDO, GREIFENSEE, SWITZERLAND^25^.

## Results

**Figure 1:**
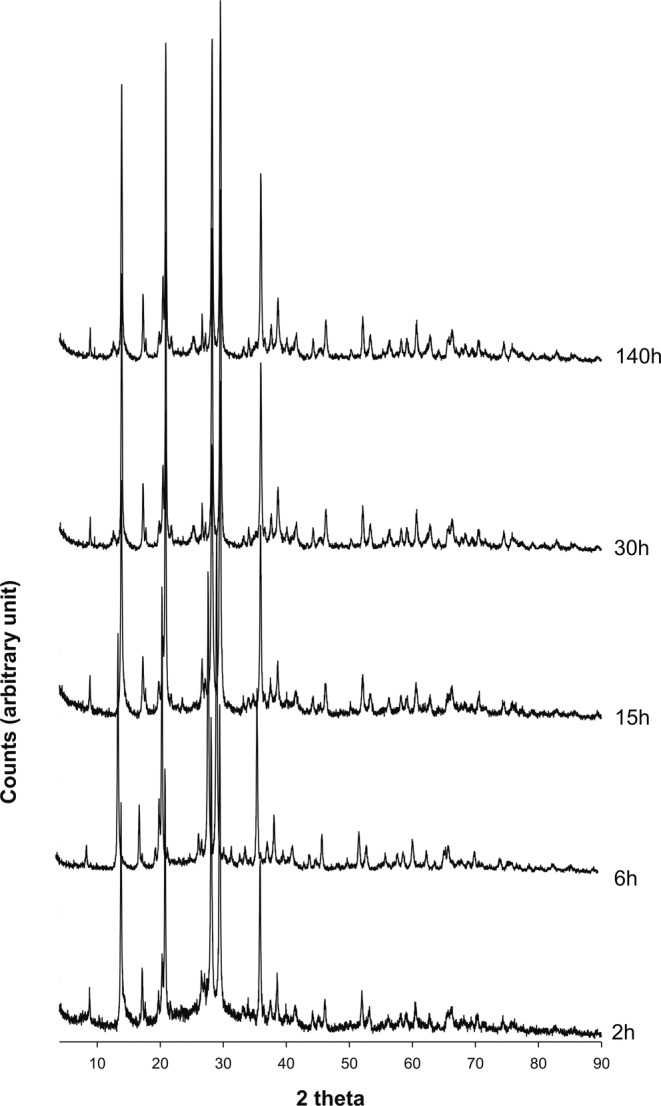
X-ray diffractometric sequence of the synthesis run.

Results of XRPD analyses performed on the synthesis run are illustrated in Fig. 1. Appearance of Li-ABW phase begins at about 2 h. The existence field of the Li-A(BW) zeolite is very large, in fact the phase remains isolated up to 140 hours.

**Figure 2:**
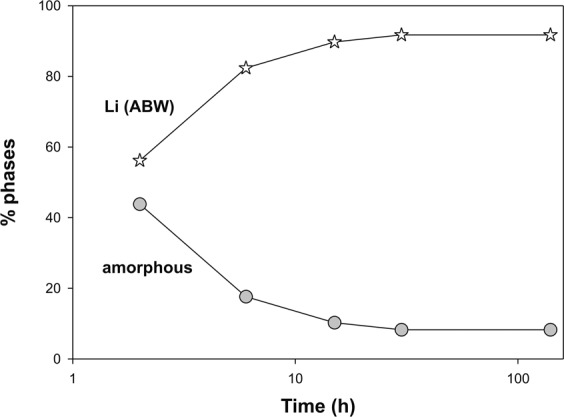
Weight fractional changes of phases as a function of time.

Results of the QPA analyses conducted on samples at 2, 6, 15, 30 and 140 h are reported in Table 1. Figure 2 illustrates the volume or weight fractional changes in the participating phases as a function of time. About 50% of the crystallization of the Li-ABW zeolite takes place in the first two hours of the synthesis run. The zeolitic percentage increases over time to the detriment of the amorphous component and reaches its climax at 30 hours. In fact, subsequently the trend of the graph becomes horizontal up to 140 hours. So the climax in the crystallization of the zeolite is reached at 30 h and the sample is dominated by the presence of Li-A(BW)zeolite (91.75%).

**Table 1:** Results of the QPA analyses conducted on samples at 2, 6, 15, 30 and 140 h.

sample	2h	6h	15h	30h	140h
Wavelenght (Å)	15.418	15.418	15.418	15.418	15.418
No. of observation	7574	7543	7428	7857	7605
R_wp_	0.15	0.15	0.19	0.18	0.17
Rp	0.11	0.11	0.14	0.14	0.13
CHI^2^	1.78	1.64	2.51	2.41	2.23
% amorphous	43.82 (11)	17.64 (8)	14.24 (13)	8.25 (7)	8.25 (17)
% Li-ABW	56.17 (14)	82.36 (8)	85.75 (18)	91.75 (9)	91.75 (14)

**Figure 3:**
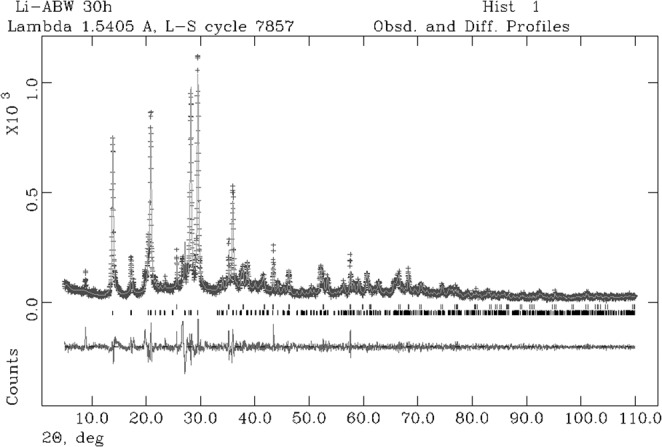
Rietveld refinement plot: Observed (+) and calculated profiles and difference plt for Li-A(BW) zeolite and corundum NIST 676a with tick marks at the position of the Bragg peaks. From the bottom: Li-A(BW) zeolite, curundum NIST 676a.

For the sample at 30 h the observed and calculated profiles and difference plots for LiABW and corundum NIST 676a are reported in Fig. 3. Cell parameters of Li-A(BW), refined with orthorombic simmetry space group *Pna2*_1_, are reported in Table 2. Cell parameters remain constant within error as a function of the experimental run time.

**Table 2:** Cell parameters of Li-A(BW) zeolite at different time sas desumed by Rietveld analysis.

	Li - ABW	*Pna2*_1_
	*a* (Å)	10.3372 (34)
2h	*b* (Å)	8.1971 (12)
	*c* (Å)	5.0111 (18)
	*a* (Å)	10.3322 (38)
6h	*b* (Å)	8.2022 (14)
	*c* (Å)	5.0093 (15)
	*a* (Å)	10.3107 ((29)
15h	*b* (Å)	8.2013 (16)
	*c* (Å)	5.0082 (19)
	*a* (Å)	10.3104 (33)
30h	*b* (Å)	8.2018 (16)
	*c* (Å)	5.0082 (17)
	*a* (Å)	10.3124 (36)
140h	*b* (Å)	8.2012 (12)
	*c* (Å)	5.0073 (17)

**Figure 4:**
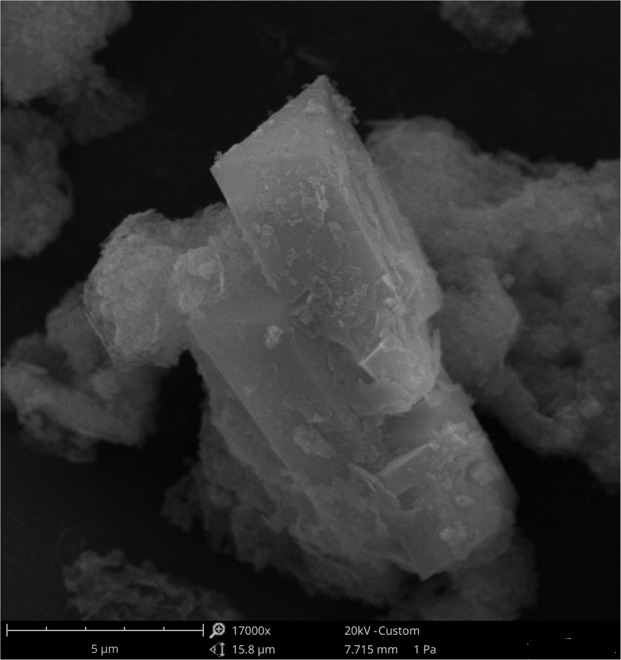
SEM image of Li-A(BW) zeolite crystal obtained at 30 h of synthesis run.

Further characterizations have been performed on the sample at 30 h. Figure 4 reports a SEM image of Li-ABW crystals from this sample with a column like morphology, with an average maximum length of crystals observed to be around 6.5 µm. Chemical analysis resulted in the stoichiometry of ^(Li^_3.98_^)(Al^_4.03_^Si^_3.98_^)O^_16_. The density of Li-A(BW) from this sample was determined to be 2.176(9) g/cm^3^, and the average volume of 0.115(9) cm^3^.

**Figure 5:**
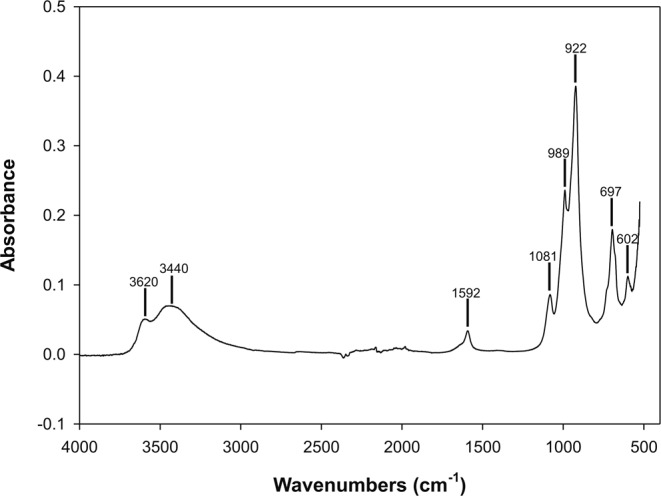
IR spectrum of the zeolite Li-ABW at 30 h.

Figure 5 illustrates the infrared spectrum of sample at 30 h. The significant broad peaks are located at 3620–3440 and 1592 cm^−1^ for O-H stretching and bending, respectively. The bands at 981, 989 and 922 cm^−1^ are assigned to the asymmetric stretching vibration of Si-O-Si bond within SiO_4_. The bands at 697 and 602 cm^−1^ are attributed to Si-O-Si symmetric stretching vibration. Data are coherent with those available in the literature^5,11^^.^

**Figure 6:**
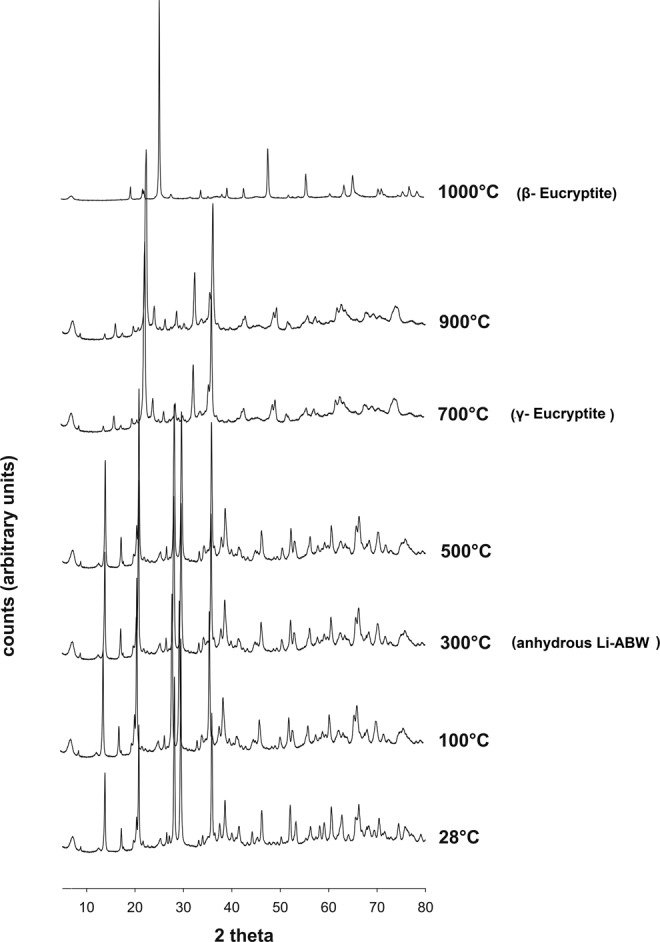
High temperature XRD pattern of sample at 30 h of synthesis run.

The study of thermal incidence on phase evolution has been completed with x-ray diffractometry at high temperature (Fig. 6). Progressive changes can be seen in the PXRD pattern of Li-A(BW) with increasing temperature and associated not only to the removal of water but also to polymorphic changes. No amorphous phases are evidenced in these transformation processes. A major change is visible in the xrd spectrum at about 300 °C which testifies the transition to anhydrous Li-A(BW). Passing from the zeolite Li-A(BW) to the anhydrous zeolite Li (ABW) we can appreciate a slight contraction of the *a*- and *c*-axes while a modest contraction of the elementary cell appears more evident for the parameter *b* passing from 8.104 Å to 7.034 Å (see Table 3). Another change in the xrd spectrum is visible at about 700 °C and related to the transition to γ-eucryptite; the space group changes in R-3. Final transformation in β-eucryptite is evidenced in the spectrum at about 1000 °C with a new change in the space group (P6422). There is good agreement with data by Norby^6^ who testifies these transformation for a Li-A(BW) prepared hydrothermally from zeolite 4 A and LiCl; the author fix the transformation into anhydrous Li-A(BW) at 300 °C, the passage at γ-eucryptite at about 650 °C and the final transformation in β-eucryptite at 900–1000 °C.

**Table 3:** Cell parameters for Li-A(BW), anhydrous Li-A(BW), γ-eucriptite and β-eucriptite.

	temperature (°C)	a (Å)	b(Å)	c (Å)	β(°)	space group
Li-ABW	28	10.347 (2)	8.104 (1)	5.011 (3)		*Pna2*_1_
Li-ABW anhydrous	300	10.012 (2)	7.034 (2)	5.007 (1)		*Pna2*_1_
γ-eucryptite	700	8.255 (2)	5.084 (1)	8.159 (3)	103.443 (2)	*R-3*
β-eucryptite	1000	10.503 (2)	10.503 (2)	11.153 (2)		*P6*_4_22

**Figure 7:**
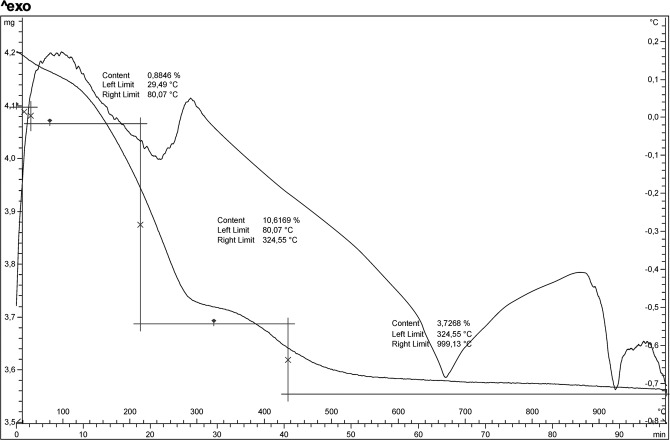
DTA-TG analysis of the sample at 30 h of synthesis run.

Thermogravimetric analysis conducted on sample at 30 h revealed a gradual and continuous water loss up to 1000 °C (Fig. 7). In particular, it indicates a three-stage mass loss. At the first stage, c.a. 0.88% loss occurred at about 80 °C; this was reasoneable due to the loss of the adsorbed and occluded water molecules in the zeolite crystals. The second stage occurred between 80° and 324 °C with a mass loss of 10,61%; the third is evidenced between 324° and 1000 °C and associated to a mass loss of 3,72%.

The endothermic peaks revealed by the DTA curve are in agreement with findings of high temperature X ray diffraction and allow the temperatures of the transformations to be fixed more precisely. The peak at about 250 °C reflects the dehydration process following the structural collapse of the phase (anhydrous Li-ABW). This is confirmed by tg measurements where at the initial weight loss of about 0.88% follows a major weight loss of 10.61% correlated to the cell contraction. Further heating of anhydrous Li-A(BW) results in the formation of γ-eucriptite at about 665 °C as visible in the endothermic peak of Fig. 6. The sharp endothermic peak at 922 °C is due to the transformation of γ-eucriptite to β-eucriptite. In our case there is an excellent correspondence between the data of the high temperature x-ray diffractometry and the thermal ones. In fact all the transformations shown by diffractometry are matched by endothermic peaks present in thermal curves. Norby^6^ for example, does not highlight the endothermic peak related to the transition from anhydrous zeolite Li-A(BW) to the γ-eucriptite. On the other hand, Yao *et al*.^11^ does not show the endothermic peak at about 900 °C, saying that the structure of γ-eucriptite is stable up to that temperature.

**Figure 8:**
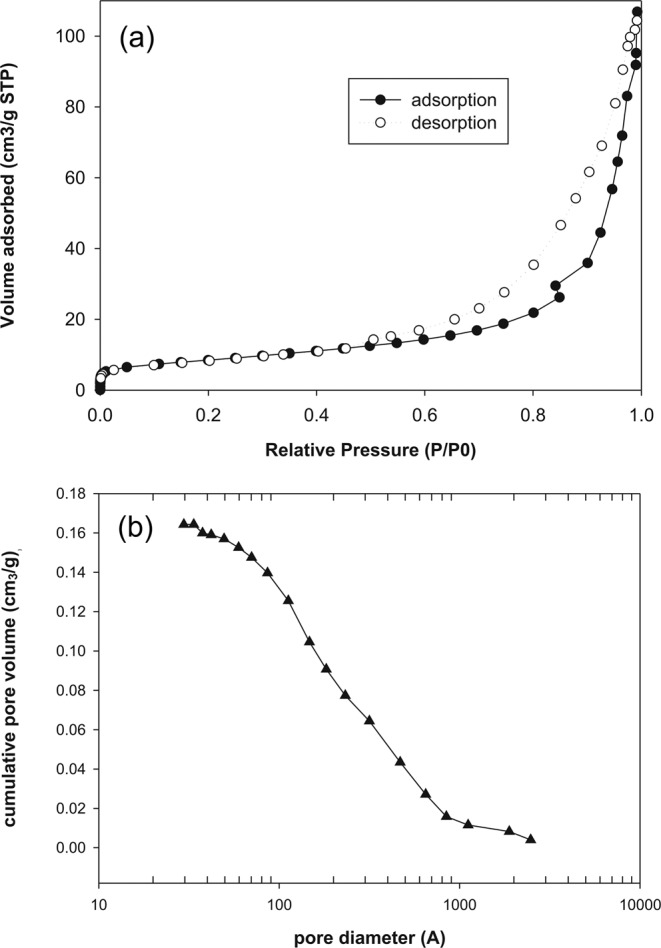
(**a**) Nitrogen adsorption-desorption isotherms of the zeolite Li-A(BW) and (**b**) corresponding pore diameter distribution pattern.

Figure 8a shows the N_2_ adsorption-desorption plots at 77 K for zeolite Li-A(BW). There is evidence of a hysteresis loop indicating the presence of mesopores; the vertical hysteresis loop indicates cylindrical mesopores, as just observed for this zeolite by Yao *et al*.^11^. The sample evidences a monomodal pore average size distribution with maximum value 29.6 Å (Fig. 8b).

## Conclusion

This work describes the synthesis of zeolite Li-A(BW) using a kaolinitic rock. Appearance of Li-A(BW) phase begins at about 2 h of the synthesis run. The existence field of the Li-A(BW) zeolite is very large, in fact the phase remains isolated up to 140 hours. An in-depth characterization of zeolite Li-A(BW) synthesized. The chemical-physical, morphological and spectroscopic characterization of experimental products proved the efficacy of the experimental procedure proposed here.

When our results are compared with those of other authors who have synthesized the same zeolite starting from a natural precursor, an improvement in the quality of crystalline products and a reduction of the calcination temperature of kaolinite, of synthesis temperature, and crystallization times is evident. Lin *et al*. in fact operate a calcination temperature of kaolinite of 800 °C, while we reduced it to 650 °C. Moreover, the same authors synthetize the zeolite at 200 °C, while in this paper there is a reduction to 180 °C. In addition, here the synthesis protocol does not include the use of additives. Another substantial difference between our work and that of Lin *et al*. lies in the effective assessment of the degree of success of the experiment from calculation by QPA of the percentage of crystallization *vs*. amorphous material and other impurities. The industry requires at least 90% pure products. And our powders reach 91.75% purity; this means that are valid results in order to try an effective transference to the productive scale. Moreover, Lin *et al*. only offers morphological and nuclear magnetic resonance characterizations for this zeolite synthesized from kaolinite. This work provides a full spectrum of physico-chemical analysis in order to characterize the synthesis products. The results of the QPA analyses and the wide temporal range of stability of this zeolite suggests that transfer to an industrial production scale would be possible.
